# Increasing the Capacity for Clinical Training of Family Medicine for Learners in a Regional Department Using Quality Improvement Tools

**DOI:** 10.7759/cureus.106231

**Published:** 2026-03-31

**Authors:** Afzal Khan, Brandon Jones, Samina Ayub, Erum Azhar, Abdul Waheed

**Affiliations:** 1 Primary Care Sports Medicine, Creighton University East Valley Arizona, Gilbert, USA; 2 Department of Family and Community Medicine, Creighton University School of Medicine, Phoenix, USA; 3 Non-operative Sports Medicine, Dignity Health Medical Group Arizona, Gilbert, USA; 4 Family Medicine, Creighton University East Valley Arizona, Gilbert, USA; 5 Family Medicine, Dignity Health Medical Group Arizona, Phoenix, USA; 6 Department of Obstetrics and Gynecology, Creighton University School of Medicine, Phoenix, USA; 7 Obstetrics and Gynecology, Creighton University East Valley Arizona, Gilbert, USA; 8 Family Medicine, Dignity Health Medical Group Arizona, Gilbert, USA

**Keywords:** family medicine clerkship, family medicine department, learner load, pa students, teaching capacity

## Abstract

Background: Clinical departments in medical schools are under increasing pressure due to ever-increasing learner load in the background of medical schools launching various initiatives to fight physician shortage in the country. A regional family medicine department sought to boost its teaching capacity for learners from an academic partner. Historically, only a few clinical sites within the department engaged in teaching third-year core clerkship, fourth-year sub-internship, and physician assistant (PA) students. The main objective was to enhance the department's teaching capabilities by transforming clinical care and provider training by leveraging Quality Improvement (QI)/Process Improvement (PI) tools to increase the number of learners per clinical Full-Time Equivalent (cFTE) per month.

Methods: QI/PI tools of process mapping, fishbone exercises, and stakeholder analysis were used to create a multifaceted bundled intervention in this quasi-experimental study. It addressed system design, external factors, and change management. Statistical Process Control (SPC) Shewhart charts were employed to track the increasing number of learners per 10 cFTE per month over time. A phase analysis was conducted to evaluate any shifts or drifts in the process using JMP Pro 19 (SAS Institute Inc., Cary, NC, USA). Further, Poisson regression was used for inferential statistics with a significance at p-value less than 0.05.

Results: The Individual Range (IR) control chart indicated a significant rise in learners, from an average of 4.7 per month at baseline to 16.4 per month post-intervention. Phase analysis of the U-Shewhart chart revealed a substantial shift in the process from baseline with the average completion rate of learners per 10 cFTE increasing from 2.5 to 7 post-intervention. Poisson regression analysis showed a statistically significant difference across the three phases (p-value <0.0001).

Conclusion: A multifaceted bundled intervention was associated with an increase in capacity to train medical and PA students in a regional family medicine department. QI/PI tools have potential for utilization in medical education.

## Introduction

Healthcare in the United States is undergoing significant workforce shortages [1]. Physician workforce shortage is specifically projected to worsen over the next decade. One prediction model by the Association of American Medical Colleges (AAMC) projected that the United States will be short of about 86,000 physicians by the year 2036 [2]. Many different strategies have been suggested to deal with this current as well as the projected shortage in future. Most of these strategies include components like 1) Increasing the class sizes and enrollment in currently Liaison Committee on Medical Education (LCME) and Commission on Osteopathic Colleges Accreditation (COCA) accredited medical schools; 2) Addition of new accredited medical schools; 3) “Supplementing” with non-physician providers like physician assistants (PAs) and nurse practitioners; and 4) Utilization of pipeline of international medical graduates (IMGs) through Graduate Medical Education (GME) or some other “GME bypass” pathways [3,4].

With all this growth pressure, many accredited medical schools are increasing enrollment and class sizes. This has been reported to increase the demands on clinical teaching departments at medical schools and their aligned health systems [5]. The Association of Departments of Family Medicine (ADFM) noted the need for increasing the capacity of clinical family medicine teaching for additional learners, such as advanced practice practitioner (APP) students. The number of APP students has really grown in the past decade or so [5]. Along with that, the increase in class size in the medical school brings an even more unique challenge as the departments try to provide decentralized clerkships. Departments of family medicine often have disproportionately large and valuable teaching roles in early clinical skills development [5]. This is in addition to already depleting financial resources and multiple other competing interests in practice transformation [6]. While these challenges are there, the ADFM urged all-hands-on-deck to utilize such opportunities to advocate for the discipline of family medicine and stoke interest for the specialty among more medical students [5]. Nevertheless, an increase in capacity for more learners in the clinical environment requires strategic planning and tactical work to recruit, develop, and retain faculty [7]. There is a considerable cost to family medicine clerkship. It is expected to increase with the increased class size [8,9]. This gets even more complex when health systems hire the faculty for medical school clinical clerkships. The increasing cost must be contained and managed with the context of clinical work. There is an increasing trend among health systems that medical groups supporting medical school faculty assume the clinical teaching during patient-facing hours for granted [9]. This calls for innovative solutions with a cohesive approach to clinical care and teaching during patient-facing hours. This study evaluated the impact of a multi-faceted bundled intervention in building a cohesive approach towards to increase capacity in a regional department of family medicine as measured by the number of learners per clinical Full-Time Equivalent (cFTE) per month.

## Materials and methods

Study design and setting

This is a quasi-experimental study with a pre and post design to evaluate the outcomes of a multifaceted bundled intervention to increase the capacity to teach the learners in a regional academic family medicine department. It is part of a larger educational endeavor to bring five family medicine clinic sites together to create a cohesive regional department of academic family medicine. Dignity Health, Arizona and Creighton University School of Medicine and Health Sciences Campus in Phoenix are part of an alliance to train medical students (MS) and other health professionals, including students of Master of Physician Assistant Studies (PA students) [10]. The five family medicine clinic sites in the department are situated in different neighborhoods of the greater Phoenix area in the state of Arizona.

This study followed the SQUIRE 2.0 Guidelines [11] for quality assessment and improvement results. The study did not involve the collection of any patient-facing data. The analysis involved de-identified, aggregate data of number of students, physicians, and APPs in different clinic sites in the department. The data were initially collected for educational improvement purposes and no additional data was collected for this study. The Dignity Health Arizona Research Enterprise (DHARE) Institutional Review Board (IRB) approved it as Not Human Subjects Research (NHSR) (notification EVR-NR-26-510-097-73-47).

Pre-intervention state

**Figure 1:**
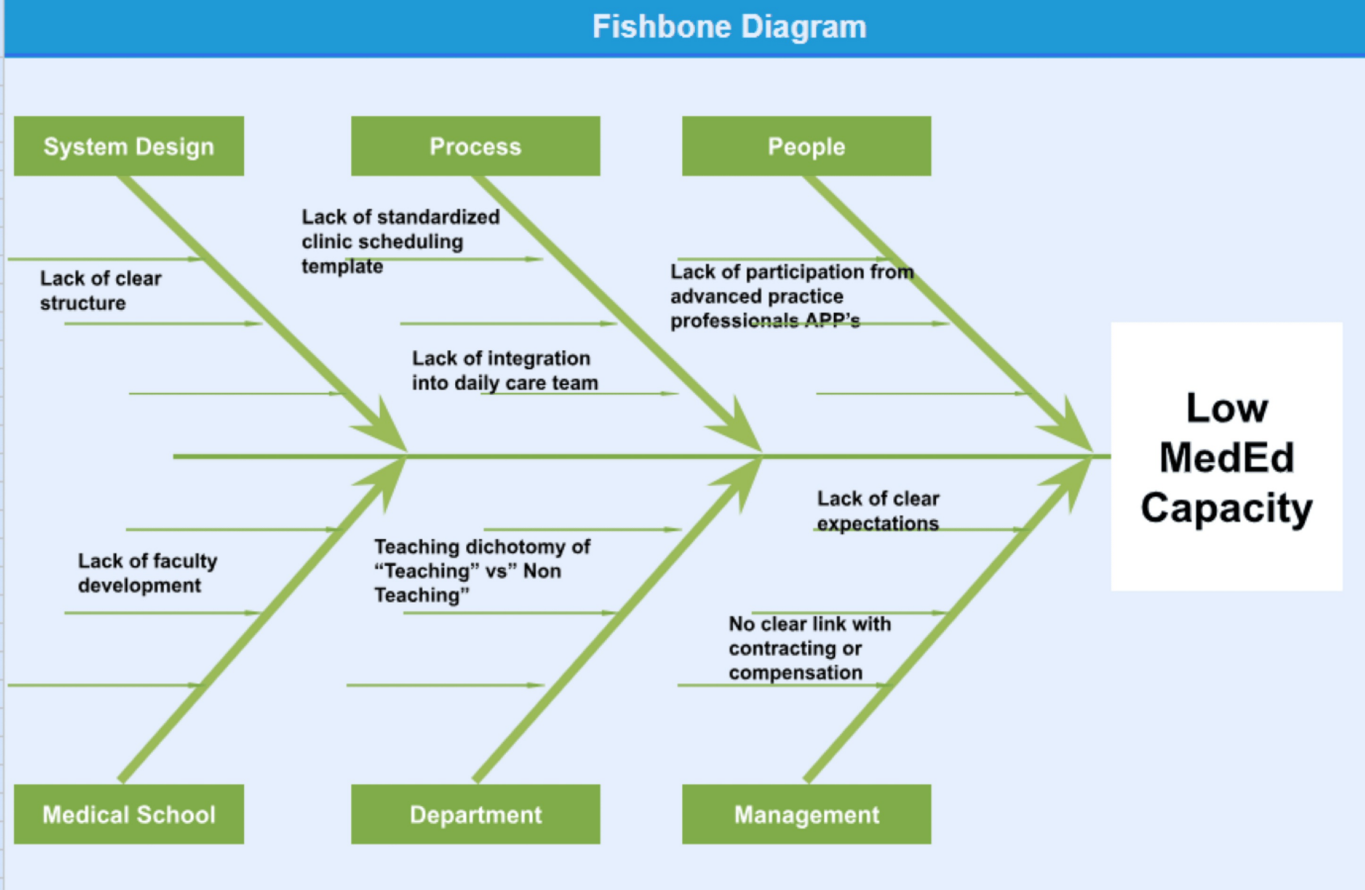
Ishikawa diagram explaining the root causes of low MedEd (teaching) capacity in the department MedEd capacity: Medical education capacity or capacity to hold learner load; APPs: advanced practice practitioners

Department Chair, Director of Operations, and members of the study team performed an Ishikawa exercise to determine the root causes contributing to low capacity for clinical family medicine teaching in the department. The analysis detailed factors in system design, process of daily operations, and human resources. The Ishikawa or Fishbone diagram from this exercise is shown in Figure 1.

Similarly, a detailed stakeholder analysis was performed to devise strategies to influence and change management. A summary of the stakeholder analysis is shown in Table 1. The study team also included an improvement advisor from a different department who is trained and is a Certified Professional in Patient Safety (CPPS) as well as a Certified Professional in Healthcare Quality (CPHQ).

**Table 1:** Key stakeholder analysis and influence strategies MS: medical student; PA: physician assistant

Key Stakeholder	Challenge or Concern	Influence/Win	Influence Strategy
Institutional Leadership	Ensuring that institutional priorities across the partner institutions, Creighton University and Dignity Health, is aligned similar to their missions	Investment into full alignment of institutional missions and shared institutional goals resulting from the partnership in the Alliance	Engagement at institutional leadership forums, direct 1:1 meetings with the Regional Dean and Dean’s cabinet in Phoenix as well engagement with the C-suite executives at Dignity Health Medical Group, AZ
Faulty/Department Leadership	Lack of leadership structure in the department Lack of clear expectations and participation in teaching at different sites	Inherent and intrinsic drive to teach, meaning and Joy in medicine, personal/professional fulfilment Addition of teaching in the “Value” metrics of compensation	Engagement at the regularly scheduled clinic provider meetings Customized and catered faculty development program, alignment of end-of-the year compensation bonus with related to value metrics
Students	Lack of familiarity with “newer” sites of clinical teaching Driving distance from the school campus and “newer” sites of clinical teaching	Desire to obtain skills and experience that allow them to pass the shelf exam, obtain good grade in the rotation, and become excellent professionals	Proactive engagement and provision of information on available clinic sites, help the faculty with improvement of the care provision and documentation.
MS Clerkship & PA Program Leadership	Lack of engagement and coordination with previous leadership	Intrinsic desire and responsibility/investment in to making their programs successful	Regular strategic planning meetings as well as operational check-ins between the program leadership and departmental leadership and administrative personnel

The intervention

**Figure 2:**
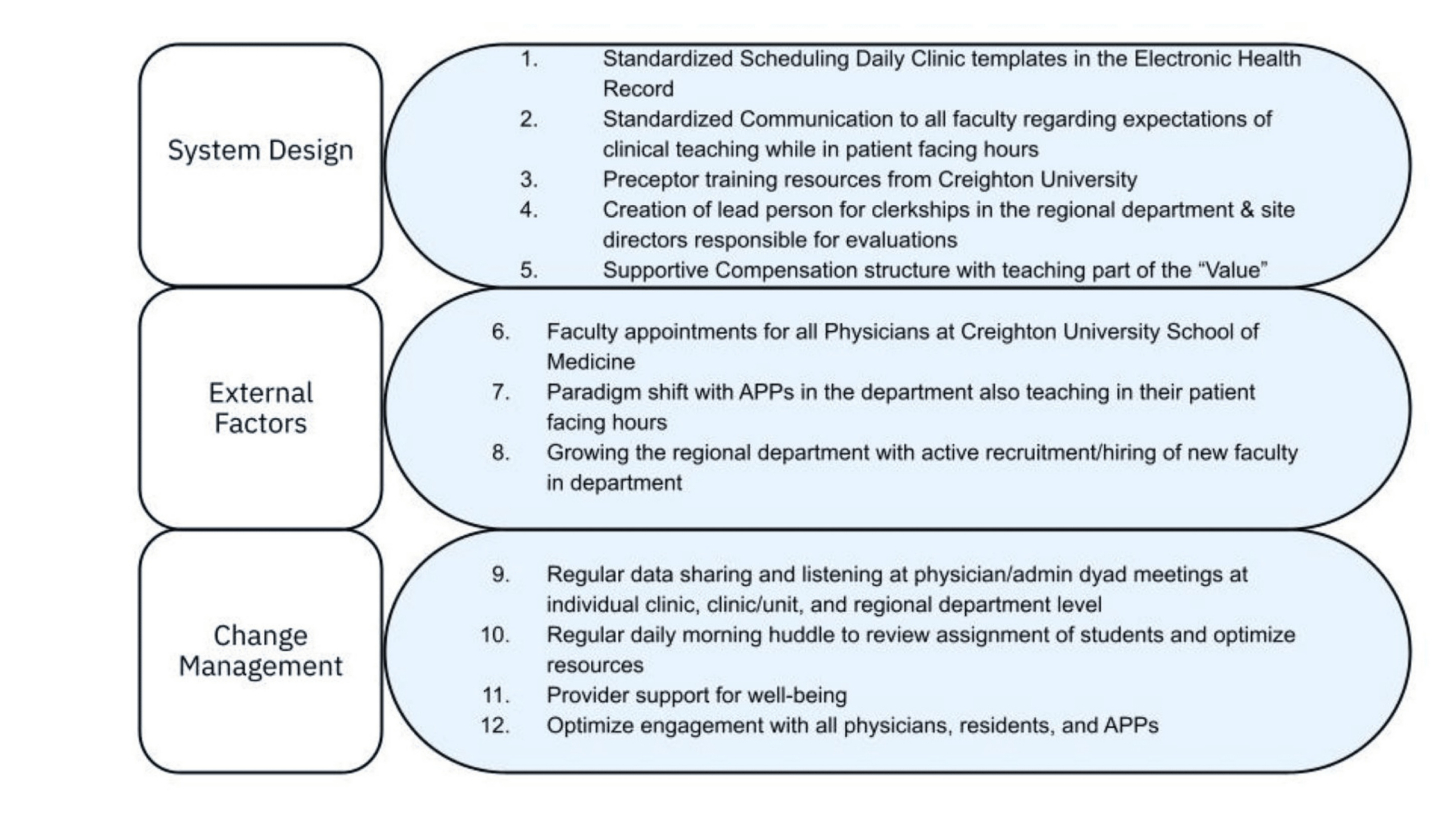
Elements of multifaceted interventional bundle APPs: advanced practice practitioners

The present study introduced a multifaceted intervention built on the elements learnt from the Ishikawa exercise and stakeholder analysis. It was aimed at increasing the number of learners per month per 10 cFTEs. It had three domains: 1) System Design, 2) External Factors, and 3) Change Management. The details of the elements of the intervention bundle are shown in Figure 2.

Data collection and analysis

The data was collected on the number of learners per month in each of the clinic sites along with the number of cFTEs on the site. The learners included students requiring clinical teaching for their third year Core Family Medicine Clerkship (MS-3s), fourth year Family Medicine Sub-Internship/Audition/Elective rotation (MS-4s), and students of Physician Assistant Studies (PA students). The Dignity Health Medical Group utilizes distribution of Full-Time Equivalent (FTE) of every individual clinician across their FTE into CARTS: Clinical, Administration, Research, Teaching, Strategic/Service. The dedicated time/FTE distribution outside of CARTS requires significant time required to do activities in each domain outside of the patient-facing work. For the purpose of this study, the number of cFTEs of all physicians and APPs per month were also collected as the numerator, with the number of learners being the denominator. A ratio metric based on the number of learners per 10 cFTEs per month was built and monitored over time. Statistical Process Control (SPC) was used to display and monitor this metric over time. The individual numbers were plotted on an Individuals (XmR) chart while Shewhart’s U-control chart was used to plot the ratio metric in JMP® Pro 19 (SAS Institute Inc., Cary, NC, USA). Institute for Healthcare Improvement (IHI) special-cause rules were applied to detect non-random variation (e.g., shifts, trends) and to recalculate centerlines/control limits when indicated. Finally, for phase-level comparisons, we conducted Poisson regression analysis keeping α = 0.05.

## Results

The department continued to grow over the study period. At the outset of the study period, there were total of 11 physicians and seven APPs in five clinical sites. These increased to 21 physicians and nine APPs as shown in Table 2. The numbers of physicians and APPs in the regional department with their clinic sites at the conclusion of the study are shown in Table 2. Consequently, the cFTE distribution across all clinical sites also grew over the study period. The number of learners per month also increased over the study period.

**Table 2:** Physicians and advanced practice practitioners (APPs) and their care sites in the regional department DHMG: Dignity Health Medical Group

Clinic Site	Number of Physicians	Number of Advanced Practice Practitioners or Residents
DHMG Family Medicine, St. Joseph Hospital (Peppertree)	6	3 APPs
DHMG Family Medicine, Ahwatukee	2	2 APPs
DHMG Family Medicine, Chandler	2	2 APPs
DHMG Family Medicine, Gilbert	8	18 residents (6 per year in a 3 year program)
DHMG Family Medicine, Queen Creek	3	2 APPs

**Figure 3:**
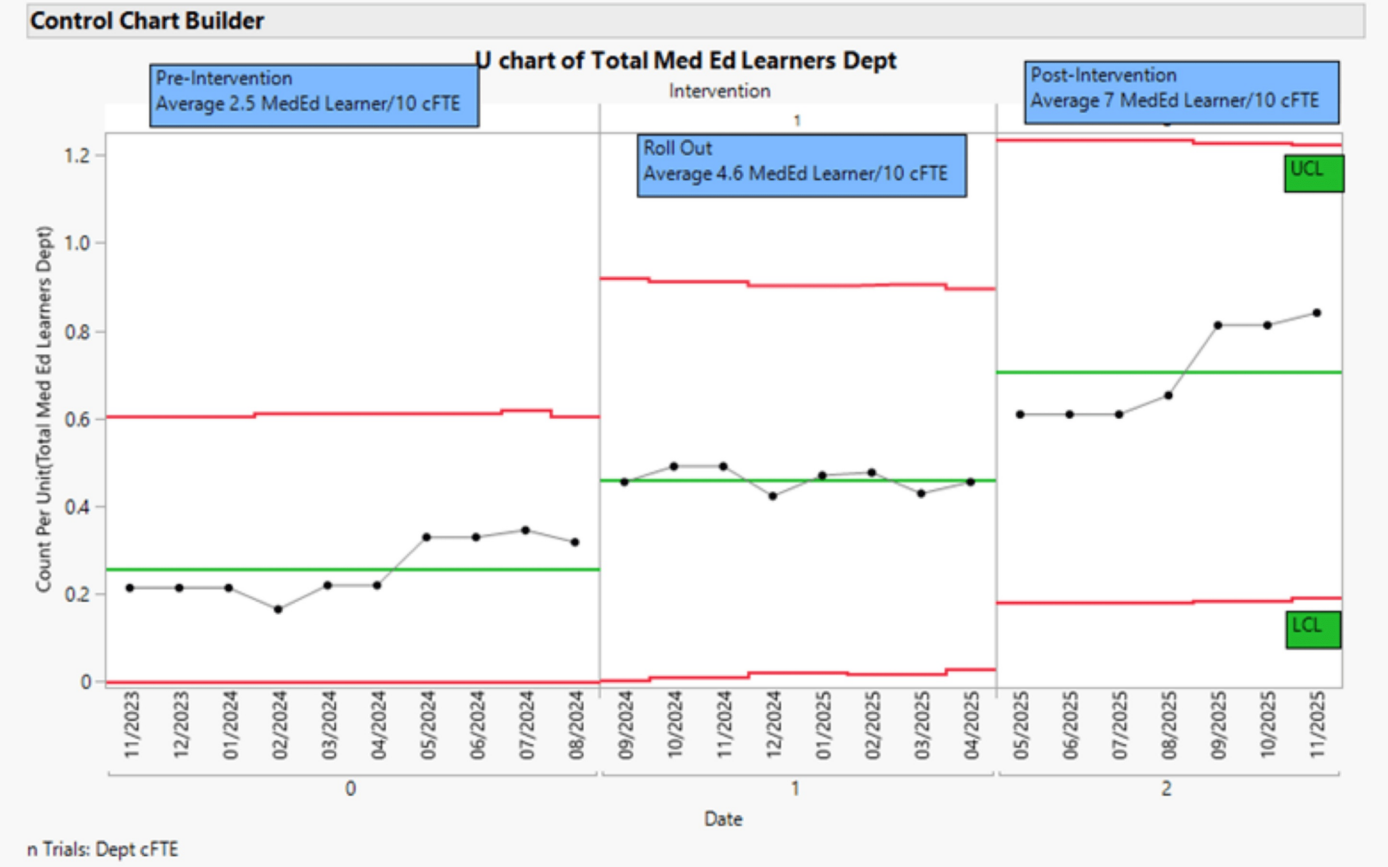
Three-phase analysis of U-control chart of the number of learners per 10 clinical Full Time Equivalent (cFTE) per month. X-axis: Date of measurement, Y-axis: number of learners per cFTE per month, Pre-intervention (0), Rollout period (Phase 1) and Post-intervention (Phase 1) separated by the (grey) intervention line. μ0 represents the mean as the center green line. Upper red line: three sigma upper control limit (UCL). Lower red line: three-sigma lower control limit (LCL). Values in UCL and LCL represent three standard deviations above and below the mean, respectively. Special cause variation is highlighted in red with the Nelson rule number 1 and a change in the mean from the pre-intervention to the post-intervention phase.

The Individual Range (IR) control chart indicated that the total number of learners in the department increased from an average of 4.7 per month at baseline to 16.4 per month after the implementation of the intervention. Based on that, a three-phase analysis of the number of learners/10 cFTEs per month was built and is shown in Figure 3. It shows a clear shift in the process by the Institute for Healthcare Improvement (IHI) rules for special cause determination and for detection of shifts and drifts in the process.

Rollout of the multifaceted bundled intervention was initiated in August 2024, marked as Phase 1 or Rollout phase. In this phase, the number of learners per 10 cFTEs increased 80.8% from baseline. The distribution of points after August 2024 fulfilled the special cause rule for a shift, as defined by the IHI, prompting a recalculation of the centerline and the designation of the period from August 2024 through April 2025 as a shift. After the bundled intervention was fully implemented, a second significant increase in average baseline measurements demonstrated rule-based evidence of special cause, denoting a new shift marked in the post-implementation phase (Phase 2). In this period, the number of learners per 10 hours of cFTE per month rose 152.2%. When compared to pre-intervention, this represents an increase of 280.0%. Just by glance, there is a visible increase in September of 2025, which was sustained afterwards at the conclusion of the study. That marks an increased number of learners with the new PA class getting into clinical learning environments with a befitting response in the data plot from the department. Overall, the analysis here shows a remarkable increase in the number of learners trained in the department. This indicates a successful increase in the teaching capacity (MedEd capacity) in the department.

**Figure 4:**
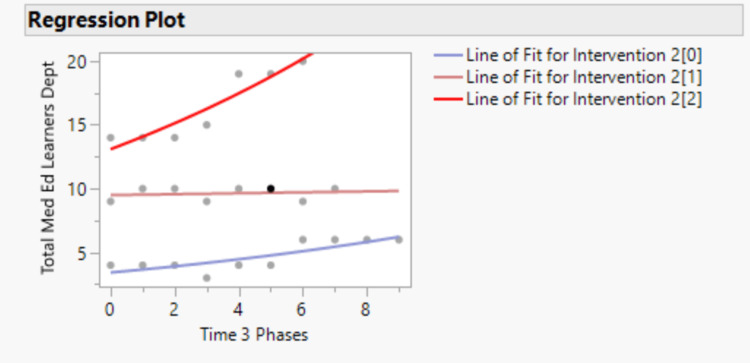
Poisson regression plot of total number of learners throughout the department across three phases of intervention and time

Further, Poisson regression analysis was performed to detect statistically significant differences in the number of learners in the department per month after adjusting for the number of cFTEs. Figure 4 shows the Poisson regression plot. It demonstrates a differential association between time and the total number of medical education learners across phases of intervention.

The distribution of number learners with respect to intervention phase (Line of Fit for Intervention 2(0)) exhibited low baseline learner counts with a modest positive temporal trend, indicating minimal growth over the study period. The distribution of number of learners with respect to the post-intervention phase and rollout phase (Line of Fit for Intervention 2(1)) was associated with moderate baseline counts and a near-zero slope over time, suggesting relative stability in learner volume without significant temporal change. In contrast, the distribution of itself (Line of Fit Intervention 2(2)) showed the highest baseline learner counts and a pronounced positive time effect, reflecting a substantial increase in expected learner counts across phases. The nonparallel slopes across intervention levels indicate a significant time-by-intervention interaction, with time exerting a stronger multiplicative effect on learner counts with the rollout and implementation of the intervention. Collectively, these findings are shown in numbers in Table 3.

**Table 3:** Poisson regression analysis of the number of learners in the department at baseline (Phase 0), intervention rollout (Phase 1), and post-implementation of the intervention (Phase 2).

	Parameter Estimate	Standard Error	Wald ChiSquare	p-value
Intercept	1.23	0.29	17.17	<0.0001
Intervention Phase 1 versus Phase 0	0.78	0.19	15.89	<0.0001
Intervention Phase 2 versus Phase 1	0.58	0.15	14.97	0.0001

Figure 4 and Table 3 together suggest that higher-intensity intervention exposure is associated with both greater baseline capacity and accelerated growth in medical education learners over time. This difference in the three phases is statistically significant (p-value <0.0001). Together, the SPC signals and Poisson regression results support the interpretation that this multifaceted bundle was associated with significant increase in the learning capacity in a regional department of family medicine.

## Discussion

Ultimately, this study demonstrates that this bundled intervention was successful at increasing the number of students who were able to be accommodated in one family medicine department, as demonstrated by the measure of learners per 10 cFTE of faculty time. This bundled intervention consisted of 12 smaller interventions (see Figure 2) and showed a 280% increase in learners per 10 cFTE within the two-year period ending in November 2025.

This is a significant result for multiple reasons. Not in the least, this is a boon to the efforts of medical education in the area studied. However, it also shows that significant gains can be made in this area despite frequently cited fears of difficulty in finding sufficiently high-quality rotation sites for clerkships [12] as clerkship and program directors’ anxiety regarding number of students requiring placement fails to decrease [13,14]. Increasing volumes of learners, as well as increasing difficulty in obtaining high-quality rotation sites has been a concern since at least 2015 [15]. Further shifts in the economic and educational landscape in the United States, including difficulty procuring funding for academics [16], the COVID-19 pandemic [14], and other disrupting events as previously outlined, have resulted not only in an explosion of learners requiring primary care sites for rotations, but concern for increased difficulty in finding qualified sites [16]. At the height of the COVID-19 pandemic, one survey showed that 87% of medical school deans were concerned that they would have difficulty finding sufficient sites for primary care rotations for all of their learners [14]. The fact that this bundled intervention was able to increase the number of students one system could accommodate by almost threefold is noteworthy.

The reasons for the success are likely to be many and varied. Individual physicians have many different motivations for accepting students, and it is likely that no single intervention will be as effective as a multi-pronged approach. Porter noted in 2014 that while one chief concern of many providers in taking students is that it may affect the financial viability of their practice, many physicians find taking on learners beneficial in other ways [12]. Providers who teach are often better motivated to stay up to date on information likely to come up in discussions with a learner, and students may bring knowledge of new medical advances that the provider was not previously aware of. Some providers may feel an obligation to advocate for the specialty by showing new students why primary care can be a rewarding career. Still others simply recognize that it took an immense number of resources to train them and feel a moral imperative to pay that effort forward to the next generation of physicians [14]. When healthcare systems implement multiple policies to encourage these diverse impulses on the part of their providers toward educational involvement, they may be more likely to meet with success.

Some notable strengths of this study include a high level of statistical significance and a large size effect in the form of the association of increase in learners per cFTE with the implementation of a multifaceted bundled intervention. These results also translate into a higher number of learners exposed to high-quality primary care rotation sites, which may result in greater retention of the students and primary care residencies and jobs after graduation [17]. Despite the strengths, this study has several limitations. Given the type of methodology, it cannot tease out which factors from the bundled intervention were the most statistically significant. Using this method, it is impossible to say whether specific interventions within the intervention bundle were more closely linked to the primary outcome than others, or if there are synergistic or detrimental effects when considering the combinations of these interventions. The use of modern quality and process improvement tools offers real-life pragmatic evidence for improvement and can only contribute to the conclusion of associations and not causality. Future studies could utilize a more specific approach to compare the relative benefits of individual interventions or potentially analyze how interventions affect systems that start out above or below two learners per 10 cFTE.

## Conclusions

The department of family medicine in this study had a mismatch in the demand of clinical training and existing teaching capacity. This was a result of increase in the medical student enrollment and increased class size as well as the start of a new program for PA studies. This study shows that a multi-faceted bundled intervention built on Ishikawa diagram exercise, stakeholder engagement, and analysis was associated with a significant increase in one system’s capacity to take on and teach more learners in a regional family medicine academic department. Although not typically used in medical education, modern QI/PI tools like Ishikawa exercise, process mapping, building and rolling out multi-faceted bundled intervention, and SPC charts present immense potential for extensive utilization in medical education. Further improvements require a targeted approach, analyzing clinics/regions above and below average in terms of learner capacity.
